# Immunotherapy and Chemotherapy Versus Sleep Disturbances for NSCLC Patients

**DOI:** 10.3390/curroncol30020155

**Published:** 2023-02-06

**Authors:** Paul Zarogoulidis, Dimitrios Petridis, Christoforos Kosmidis, Konstantinos Sapalidis, Lila Nena, Dimitrios Matthaios, Konstantinos Porpodis, Paschalis Kakavelas, Paschalis Steiropoulos

**Affiliations:** 1 Pulmonary Department, General Clinic Euromedica Private Hospital, 851 05 Thessaloniki, Greece; 2 3rd Surgery Department, AHEPA University Hospital, Aristotle University of Thessaloniki, 541 24 Thessaloniki, Greece; 3 Department of Food Technology, School of Food Technology and Nutrition, Alexander Technological Educational Institute, 574 00 Thessaloniki, Greece; 4 Laboratory of Social Medicine, Medical School, Democritus University of Thrace, 691 00 Alexandroupolis, Greece; 5 Oncology Department, General Hospital of Rhodes, 851 00 Rhodes, Greece; 6 Pulmoanry Department, G. Papanikolaou General Hospital, Aristotle University of Thessaloniki, 541 24 Thessaloniki, Greece; 7 Intensive Care Unit (ICU), General Clinic Euromedica, 153 43 Thessaloniki, Greece; 8 Department of Respiratory Medicine, Medical School, Democritus University of Thrace, 691 00 Alexandroupolis, Greece

**Keywords:** lung cancer, cancer, chemotherapy, sleep disturbances, fatigue, insomnia, polysomnography

## Abstract

Introduction: Cancer patients are known to experience sleep disturbances that differ between disease stages and treatments. Regarding lung cancer patients and immunotherapy, information on their sleep disturbances has been recently acquired, but no comparison has been made between different treatment modalities. Patients and Methods: We recruited 98 non-small cell lung cancer patients; 49 had programmed death-ligand 1 expression of ≥50% and received immunotherapy as first-line treatment and 49 had programmed death-ligand 1 expression in the range from 0–49 and received chemotherapy as first-line treatment. All patients were stage IV, but with no bone metastasis. Sleep disturbances were recorded through polysomnography and sleep questionnaires. Results: For immunotherapy patients with PD-L1 expression ≥ 50%, the disease response was rapid and the sleep disturbances decreased rapidly. On the other hand, for chemotherapy patients, the sleep disturbances remained for all those patients that had partial response and stable disease. It was noticed that chemotherapy drugs induce severe adverse effects. Discussion: In our study, it was observed that patients with complete response had reduced sleep disturbances in the case of immunotherapy patients. However, sleep disturbances continued for several patients in the chemotherapy group due to the adverse effects of chemotherapy drugs. In conclusion: Immunotherapy drugs on their own do not induce sleep disturbances and, through treatment response, alleviate sleep disturbances in lung cancer patients.

## 1. Introduction

Lung cancer patients still tend to be diagnosed in advanced stage diseases due to the lack of early-disease symptoms. We lack a unified early-disease detection program [1]. Currently, we diagnose lung cancer with radial-endobronchial ultrasound and convex-endobronchial ultrasound [2]. The method that we currently use as an imaging technique for lung cancer staging is positron emission tomography fused with computed tomography (PET-CT) [3]. Cancer patients are known to have different types or sleep disturbances [4], including insomnia, anxiety, early morning wakening, late sleep onset, prolonged nocturnal waking periods, daytime sleepiness, and unrefreshing sleep [5]. Sleep disturbances have been reported to occur in up to 50% of cancer patients in some studies [5]. It is also known that sleep disturbances are different between treatments and stages. Pain and fatigue are accentuated with sleep disturbances [6]. In the past ten years, the treatment options for non-small cell lung cancer (NSCLC) were chemotherapy and tyrosine kinase inhibitors (TKIs) [7]. However, in the past five years, immunotherapy has become the point of focus, even in patients with low programmed death-ligand 1 (PD-L1) expression (≤50%). Specific patients with PD-L1 ≥ 50% can receive immunotherapy alone as first-line treatment, whereas patients with PD-L1 ≤ 50% can receive chemotherapy plus immunotherapy [8]. Studies of lung cancer patients receiving chemotherapy and their sleep disturbances have been recorded [9]. However, there are few studies in which lung cancer patients receive immunotherapy and their sleep disturbances have been examined [10]. In our current study, we compare the sleep differences in NSCLC patients receiving either chemotherapy or immunotherapy as first-line treatment. We report the characteristics of the two groups upon diagnosis without treatment and the impact of treatment after nine months. We make comparisons of the disease progression and sleep disturbances.

## 2. Patients and Methods

Ninety-eight (88 males and 10 females) with primary lung cancer diagnoses were included in the pilot study. All patients had non-small cell lung cancer (NSCLC), either adenocarcinoma or squamous cell carcinoma; we did not include non-other specific (NOS) cancer in order to obtain a genetically homogeneous sample. In total, 28 were stage IIIb and 70 were stage IV. We obtained demographic and clinical data regarding age, sex, painkillers, and finally recorded the Eastern Cooperative Oncology Group (ECOG) performance status. Forty-nine patients included had programmed death-ligand 1 (PD-L1) ≥ 50% and were stratified in three categories: 1, 50–70; 2, 71–90, and 3, 91–100. In the statistical analysis based on previous studies, the higher the expression, the higher the survival. All patients with PD-L1 ≥ 50% received only immunotherapy either with pembrolisumab or nivolumab as first-line treatment. Forty-nine patients had PD-L1 ≤ 50% and were stratified in three categories: 1, 0–20; 2, 21–40; and 3, 41–49. These patients received carboplatin AUC 5.5 and paclitaxel 190 m^2^. The main exclusion criteria included the inability to understand and answer the questionnaires that were distributed for their sleep evaluation (see next section). The study was approved by the investigational review board (ID number: 30/2022) of the AHEPA hospital and the protocol was initiated upon 6 January 2021. Written informed consent was obtained from each patient before study enrollment.

### Sleep Evaluation Methodology

Patients completed a self-report questionnaires one day before and nine months after their first treatment. In order to evaluate daytime sleepiness, we used the Greek version of the Epworth Sleepiness Scale (ESS) [11]. We used the ESS to assess daytime sleepiness over the previous three months in eight usual circumstances. The ESS was evaluated for the Greek population and the cut-off point, indicating excessive daytime sleepiness, was set at 10. We used the Greek version of the Pittsburgh Sleep Quality Index (PSQI) in order to assess sleep quality [12]. The PSQI is composed of 19 self-rated questions grouped into seven domains (subjective sleep quality [SSQ], sleep latency [SL], sleep duration [SDU], habitual sleep efficiency [HSE], sleep disturbances [SDI], use of sleeping medication [SM], and daytime dysfunction [DD]). In order to evaluate fatigue, we used the Greek version of the Fatigue Severity Scale (FSS) [13].

In order to objectively evaluate sleep quality, we performed overnight polysomnography (PSG) (Alice 3, Respironics), using a standard montage of electroencephalogram (EEG), electrooculogram, electromyogram (EMG), and electrocardiogram (ECG) signals, together with pulse oximetry and airflow detected using combined oronasal thermistors. The thoracic cage and abdominal motion were recorded by inductive plethysmography. EEG recordings were manually scored according to standard criteria. We used the Medical Research Council (MRC) scale in order to evaluate the presence and grade of dyspnea [14].

## 3. Results

In the subsequent study on OSA, the investigation was focused on potential effects of disease progress with chemo-(1) and immune-(2) therapy treatments (equally grouped from 98 patients) on specific questionnaire responses recorded at two time points: pre and post therapy treatments.

A repeated-measures ANOVA divided in two layers was employed: between-subject (or across-subject) effects by fitting the sum of repeated-measures columns to the model effects and within-subject effects or the effects analyzing the response function that fits differences in the repeated-measures columns (contrast response function).

Only the responses of the Epworth Sleepiness Scale questionnaire was proved to differ statistically significantly in some terms of the investigated factors.

The statistical analysis of between-subject sum effects revealed an overall significant effect on the combined categories progress*treatment (*p* = 0.022), an important reason to continue with further analysis and overall differences across time intervals (*p* < 0.001) for the two responses, in which the Epworth response declines by time intercept (Table 1 and Table 2).

The therapy treatment effect has a slightly significant effect (*p* = 0.048) on the Epworth response and thus meaningless, leading to a descending pattern: SD (11.6) < CR (7.2) < PD (6.5) < PR (5.6), the interpretation of which is of minor importance and beyond the scope of this study. The interaction between therapy type and disease progress did not produce a significant effect (*p* = 0.091).

**Figure 1:**
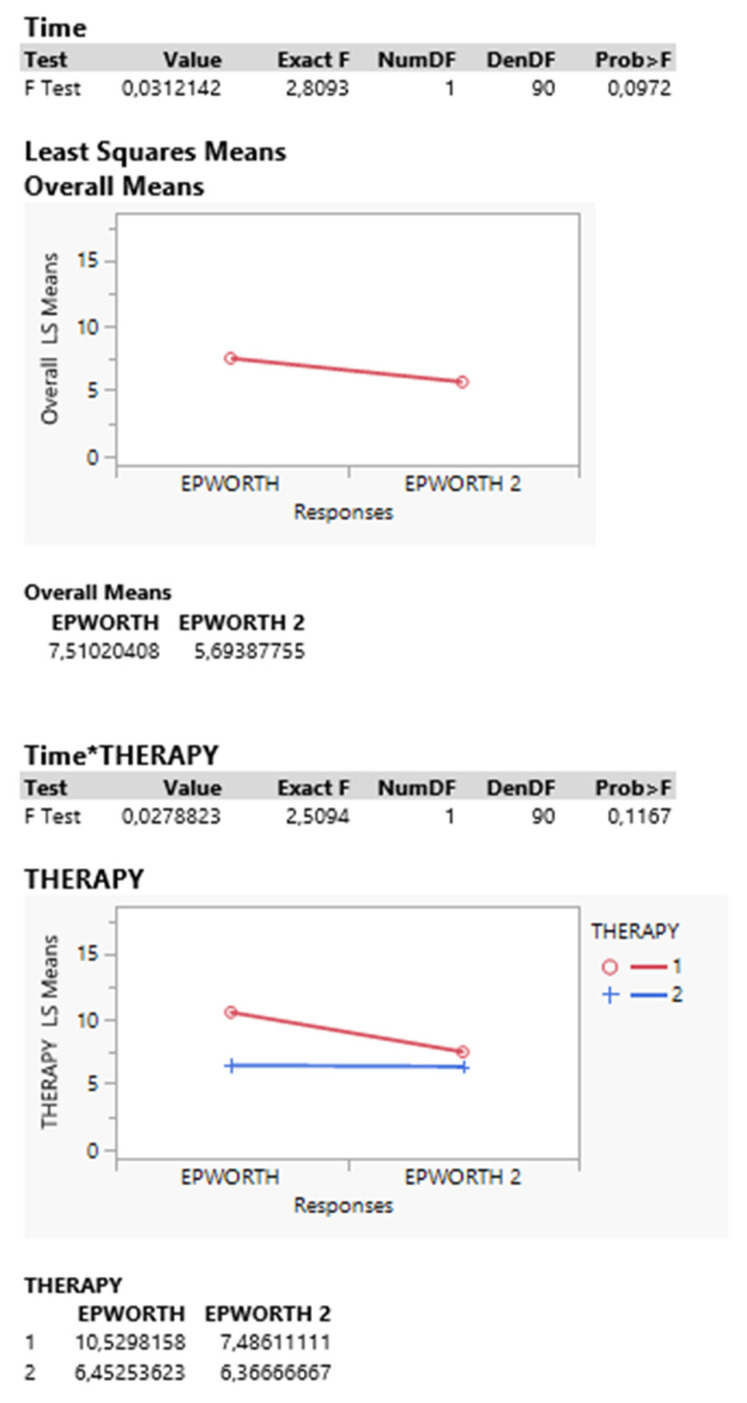
Neither interaction between time and therapy reveals a significant effect (*p* = 0.117).

The statistical analysis of within-subject effects showed a significant effect (contrast) between therapy and disease progress (*p* = 0.027), confirming that either when taking the combined sums or differences, the resulting Epworth responses are similar (Table 3 and Figure 1).

Nevertheless, the overall time interaction between responses did not produce significant effect (*p* = 0.092), although a decrease in Epworth response was observed after treatment.

**Figure 2:**
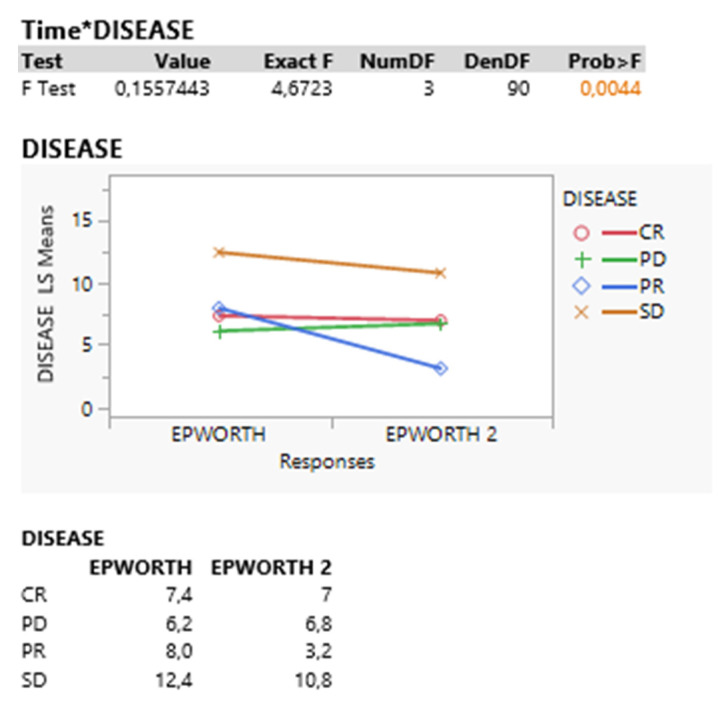
Time*disease interaction signals a significant effect (*p* = 0.004).

On the contrary, the time*disease interaction signals a significant effect (*p* = 0.004) vividly demonstrated in the following interaction plot. It appears that the main cause of disease influence derives from the decreasing SD and mainly PD physical conditions at post treatment time or Epworth response, Figure 2.

**Figure 3:**
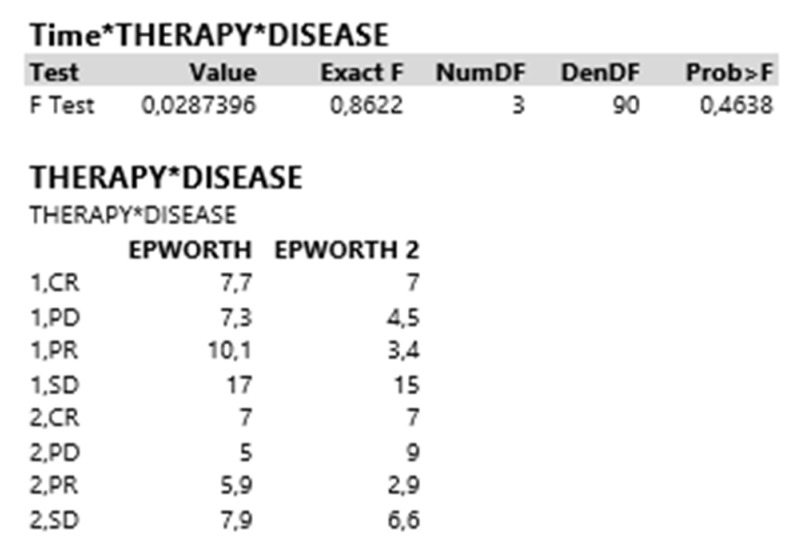
Triple interaction time*therapy*disease appeared to have an insignificant effect on Epworth responses (*p* = 0.464).

Finally, the triple interaction time*therapy*disease appeared to have an insignificant effect on Epworth responses (*p* = 0.464), Figure 3.

In conclusion, the different types of therapy did not affect the Epworth response before and after treatment in the predefined time intervals neither when combined with progressive disease. The disease progress substantially influenced the Epworth response in the post treatment period and uniquely was affected by SD or PD conditions.

## 4. Discussion

It has been previously observed that lung cancer patients have sleep disturbances upon diagnosis. These are mainly fatigue and insomnia [9]. In several cases, the fatigue is attributed to the loss of weight and insomnia due to low oxygen [15]. Moreover, chemotherapy drugs induce sleep disturbances through several mechanisms [15], whereas immunotherapy drugs have an impact on several hormones and change the metabolism. However, it has been observed that in patients with programmed death-ligand 1 (PD-L1) ≥ 50% and partial or complete disease response, these symptoms are alleviated [10]. In our study, even though several patients had complete or partial response, sleep disturbances still continued due to the drugs. Therefore, we would certainly prefer to administer immunotherapy drugs instead of chemotherapy drugs. Our study has limitations; the major limitation is the number of patients and another is that we do not have patients receiving both chemotherapy drugs and immunotherapy drugs. The reason for the second limitation is the fact that immunotherapy drugs were approved for PD-L1 expression ≤50% with the addition of chemotherapy after the protocol was approved and initiated. In our study, insomnia was alleviated in our immunotherapy group of patients with complete response unlike in other studies [10]. Furthermore, circadian rhythms were not investigated. Circadian rhythms are associated with several hormones and inflammatory agents circulating the body. Cancer patients are known to have higher concentrations of inflammatory agents in their blood. Although at the beginning of therapy, immunotherapy is a factor for increased inflammation in the body, the inflammation hormones secreted are reduced during disease response [10,16]. In contrast, chemotherapy agents maintain inflammatory agents at high levels throughout the treatment [17]. It is likely that patients receiving both chemotherapy and immunotherapy will have higher levels of inflammatory agents due to receiving two different drugs with different mechanisms of action. Fatigue is another result of this immune activation [18]. Recently, in an animal model, the overexpression of NF-kB has been identified as the common underlying factor for insomnia and inflammation [19]. Circadian clock genes play a complex role in cancer development and the anticancer immune response, regulating even the formation of tumor-related immune cell infiltrates. Thus, the ample evidence that excessive, chronic inflammatory activation may provide a link between cancer, cancer therapies, and insomnia has been addressed in a recent analysis. This issue remains to be solved in a future study with a large number of patients [20]. Upon diagnoses, the most affected component was sleep latency, followed by sleep duration. During therapy, all patients receiving painkillers stopped them due to therapy efficiency. Dyspnea is a major symptom for patients with chronic obstruction pulmonary disease (COPD) that affects both daytime function and sleep quality. However, in our study, not all our patients were smokers and the mean MRC score upon diagnosis was also low for both patient groups. Moreover, no relationship was observed between the MRC score and sleep quality in both patient groups. In our study, no correlation was observed between daytime sleepiness and sleep quality, as assessed by the global PSQI score or any of its components for both patient categories. Fatigue was observed in both groups of patients, which is associated with sleep quality with subjective sleep quality, sleep duration, and daily dysfunction. These findings are in accordance with previous studies [12,21,22,23,24,25,26,27,28,29,30,31,32,33,34]. We did not observe any differences between the two drugs (nivolumab and pembrolizumab).

## 5. Conclusions

Patients receiving chemotherapy will not experience alleviation of their sleep disturbances, in contrast to patients receiving immunotherapy. Although immunotherapy drugs induce inflammation in order to treat cancer, the balance observed during partial and complete disease response alleviates the symptoms, especially insomnia and fatigue.

## Figures and Tables

**Table 1:** All between.

**Test**	**Value**	**Exact F**	**NumDF**	**DenDF**	**Prob > F**
F Test	0.1935683	2.4887	7	90	0.0220
**Intercept**					
**Test**	**Value**	**Exact F**	**NumDF**	**DenDF**	**Prob > F**
F Test	1.5755925	141.8033	1	90	<0.0001
**EPWORTH**			**EPWORTH 2**		
8.49117603			6.92638889		

**Table 2:** Therapy.

**Test**	**Value**	**Exact F**	**NumDF**	**DenDF**	**Prob > F**
F Test	0.0447519	4.0277	1	90	0.0478
**Disease**					
**Test**	**Value**	**Exact F**	**NumDF**	**DenDF**	**Prob > F**
F Test	0.1260081	3.7802	3	90	0.0132
Therapy*Disease					
**Test**	**Value**	**Exact F**	**NumDF**	**DenDF**	**Prob > F**
F Test	0.0741014	2.2230	3	90	0.0909

**Table 3:** All within interactions.

Test	Value	Exact F	NumDF	DenDF	Prob > F
F Test	0.1867806	2.4015	7	90	0.0267

## Data Availability

The data presented in this study are available on request from the corresponding author.

## References

[1] Parekh A., Deokar K., Verma M., Singhal S., Bhatt M.L., Katoch C. (2022). The 50-Year Journey of Lung Cancer Screening: A Narrative Review. Cureus.

[2] Zarogoulidis P., Huang H., Chen W., Petridis D., Matthaios D., Hohenforst-Schmidt W., Tolis C., Tsakiridis K., Baka S., Arnaoutoglou C. (2022). Radial Endobronchial Ultrasound for Lung Cancer Diagnosis: Tips and Tricks. J. Cancer.

[3] Zarogoulidis P., Kosmidis C.S., Hohenforst-Schmidt W., Matthaios D., Sapalidis K., Petridis D., Perdikouri E.I., Courcoutsakis N., Hatzibougias D., Arnaoutoglou C. (2022). Radial-EBUS: CryoBiopsy Versus Conventional Biopsy: Time-Sample and C-Arm. Int. J. Environ. Res. Public Health.

[4] Zaric B., Stojsic V., Carapic V., Kovacevic T., Stojanovic G., Panjkovic M., Kioumis I., Darwiche K., Zarogoulidis K., Stratakos G. (2016). Radial Endobronchial Ultrasound (EBUS) Guided Suction Catheter-Biopsy in Histological Diagnosis of Peripheral Pulmonary Lesions. J. Cancer.

[5] Oikonomidou R., Petridis D., Alexidis P., Matthaios D., Boukovinas I., Perdikouri E.I., Baka S., Hohenforst-Schmidt W., Huang H., Bai C. (2022). “One Shot” Sample Evaluation of 22G, 22G upgraded, 21G and 19G needle for Endobronchial Ultrasound-EBUS-TBNA. J. Cancer.

[6] Oikonomidou R., Petridis D., Kosmidis C., Sapalidis K., Hohenforst-Schmidt W., Christakidis V., Petanidis S., Mathaios D., Perdikouri E.I., Baka S. (2022). Cryo-Biopsy versus 19G needle versus 22G needle with EBUS-TBNA endoscopy. J. Cancer.

[7] Sapalidis K., Zarogoulidis P., Petridis D., Kosmidis C., Fyntanidou B., Tsakiridis K., Maragouli E., Amaniti A., Giannakidis D., Koulouris C. (2019). EBUS-TNBA 22G samples: Comparison of PD-L1 expression between DAKO and BIOCARE((R)). J. Cancer.

[8] Zarogoulidis P., Huang H., Hu Z., Wu N., Wang J., Petridis D., Tsakiridis K., Matthaios D., Kosmidis C., Hohenforst-Schmidt W. (2021). Priority of PET-CT vs. CT Thorax for EBUS-TBNA 22G vs. 19G: Mesothorax Lymphadenopathy. J. Cancer.

[9] Zhu J., Pan F., Cai H., Pan L., Li Y., Li L., Li Y., Wu X., Fan H. (2022). Positron emission tomography imaging of lung cancer: An overview of alternative positron emission tomography tracers beyond F18 fluorodeoxyglucose. Front. Med..

[10] Young J.S., Bourgeois J.A., Hilty D.M., Hardin K.A. (2009). Sleep in hospitalized medical patients, part 2: Behavioral and pharmacological management of sleep disturbances. J. Hosp. Med..

[11] Uzer A., Kurtses Gursoy B. (2022). The mediating roles of depression, anxiety, and psychological pain in the relationship between chronotype and suicide in patients with depressive disorder. Chronobiol. Int..

[12] Spiegel D. (2008). Losing sleep over cancer. J. Clin. Oncol. Off. J. Am. Soc. Clin. Oncol..

[13] Giglio M., Preziosa A., Mele R., Brienza N., Grasso S., Puntillo F. (2022). Effects of an Intrathecal Drug Delivery System Connected to a Subcutaneous Port on Pain, Mood and Quality of Life in End Stage Cancer Patients: An Observational Study. Cancer Control J. Moffitt Cancer Cent..

[14] Zarogoulidis K., Zarogoulidis P., Darwiche K., Boutsikou E., Machairiotis N., Tsakiridis K., Katsikogiannis N., Kougioumtzi I., Karapantzos I., Huang H. (2013). Treatment of non-small cell lung cancer (NSCLC). J. Thorac. Dis..

[15] Domvri K., Zarogoulidis P., Darwiche K., Browning R.F., Li Q., Turner J.F., Kioumis I., Spyratos D., Porpodis K., Papaiwannou A. (2013). Molecular Targeted Drugs and Biomarkers in NSCLC, the Evolving Role of Individualized Therapy. J. Cancer.

[16] Domvri K., Darwiche K., Zarogoulidis P., Zarogoulidis K. (2013). Following the crumbs: From tissue samples, to pharmacogenomics, to NSCLC therapy. Transl. Lung Cancer Res..

[17] Lim J.U., Kang H.S., Shin A.Y., Yeo C.D., Kim S.K., Kim J.W., Kim S.J., Lee S.H. (2022). Investigation of poor predictive factors in extensive stage small cell lung cancer under etoposide-platinum-atezolizumab treatment. Thorac. Cancer.

[18] Economou N.T., Ilias I., Velentza L., Papachatzakis Y., Zarogoulidis P., Kallianos A., Trakada G. (2018). Sleepiness, fatigue, anxiety and depression in Chronic Obstructive Pulmonary Disease and Obstructive Sleep Apnea—Overlap—Syndrome, before and after continuous positive airways pressure therapy. PloS ONE.

[19] Kiss I., Kuhn M., Hrusak K., Buchler B., Boublikova L., Buchler T. (2022). Insomnia in patients treated with checkpoint inhibitors for cancer: A meta-analysis. Front. Oncol..

[20] Tsara V., Serasli E., Amfilochiou A., Constantinidis T., Christaki P. (2004). Greek version of the Epworth Sleepiness Scale. Sleep Breath..

[21] Kotronoulas G.C., Papadopoulou C.N., Papapetrou A., Patiraki E. (2011). Psychometric evaluation and feasibility of the Greek Pittsburgh Sleep Quality Index (GR-PSQI) in patients with cancer receiving chemotherapy. Support. Care Cancer Off. J. Multinatl. Assoc. Support. Care Cancer.

[22] Katsarou Z., Bostantjopoulou S., Hatzizisi O., Giza E., Soler-Cardona A., Kyriazis G. (2007). Immune factors or depression? Fatigue correlates in Parkinson’s disease. Rev. De Neurol..

[23] Williams N. (2017). The MRC breathlessness scale. Occup. Med..

[24] Zarogoulidis P., Steiropoulos P., Perantoni E., Archontogeorgis K., Eleftheriadou E., Porpodis K., Charpidou A.G., Angelopoulou C., Nena E., Zarogoulidis K. (2013). Subjective sleep quality in lung cancer patients before and after chemotherapy. Thorac. Cancer.

[25] Zarogoulidis P., Kosmidis C., Kesisoglou I., Tsakiridis K., Hohenforst-Schmidt W., Huang H., Romanidis K., Vagionas A., Sapalidis K. (2021). Nutrition and NSCLC; Should We Administer Food Supplements?. Curr. Pharm. Des..

[26] Zarogoulidis P., Darwiche K., Huang H., Spyratos D., Yarmus L., Li Q., Kakolyris S., Syrigos K., Zarogoulidis K. (2013). Time recall; future concept of chronomodulating chemotherapy for cancer. Curr. Pharm. Biotechnol..

[27] Seifen C., Huppertz T., Matthias C., Gouveris H. (2021). Obstructive Sleep Apnea in Patients with Head and Neck Cancer-More than Just a Comorbidity?. Medicina.

[28] Kiss I., Kuhn M., Hrusak K., Buchler T. (2022). Incidence of fatigue associated with immune checkpoint inhibitors in patients with cancer: A meta-analysis. ESMO Open.

[29] Zuo C., Hong Y., Qiu X., Yang D., Liu N., Sheng X., Zhou K., Tang B., Xiong S., Ma M. (2018). Celecoxib suppresses proliferation and metastasis of pancreatic cancer cells by down-regulating STAT3/NF-kB and L1CAM activities. Pancreatol. Off. J. Int. Assoc. Pancreatol..

[30] Zhang Z., Zeng P., Gao W., Zhou Q., Feng T., Tian X. (2021). Circadian clock: A regulator of the immunity in cancer. Cell Commun. Signal. CCS.

[31] Bower J.E., Ganz P.A., Irwin M.R., Kwan L., Breen E.C., Cole S.W. (2011). Inflammation and behavioral symptoms after breast cancer treatment: Do fatigue, depression, and sleep disturbance share a common underlying mechanism?. J. Clin. Oncol. Off. J. Am. Soc. Clin. Oncol..

[32] Mystakidou K., Parpa E., Tsilika E., Pathiaki M., Patiraki E., Galanos A., Vlahos L. (2007). Sleep quality in advanced cancer patients. J. Psychosom. Res..

[33] Mystakidou K., Parpa E., Tsilika E., Pathiaki M., Gennatas K., Smyrniotis V., Vassiliou I. (2007). The relationship of subjective sleep quality, pain, and quality of life in advanced cancer patients. Sleep.

[34] Vena C., Parker K., Allen R., Bliwise D., Jain S., Kimble L. (2006). Sleep-wake disturbances and quality of life in patients with advanced lung cancer. Oncol. Nurs. Forum.

